# Q&A - Global challenges of epilepsy management - an interview with Gretchen Birbeck

**DOI:** 10.1186/1741-7015-11-70

**Published:** 2013-03-14

**Authors:** Gretchen Birbeck

**Affiliations:** 1 International Neurologic & Psychiatric Epidemiology Program, Michigan State University, 909 Fee Road, #324 West Fee Hall, East Lansing, MI 48824, USA

## Introduction

**Figure 1:**
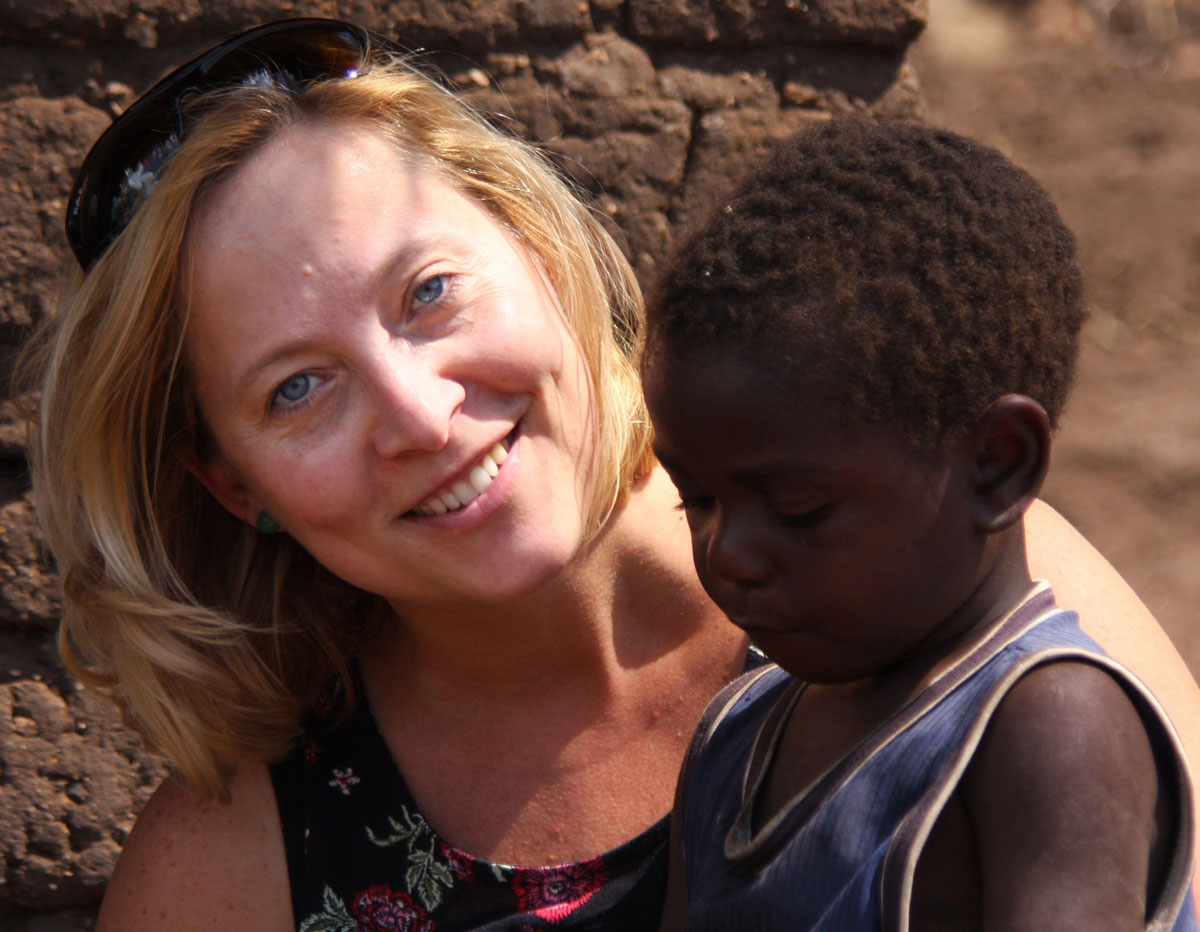
**Gretchen Birbeck**.

Gretchen Birbeck is a Professor of Neurology and Epidemiology at Michigan State University. She has roles in clinical neurology, epidemiology and health services research with a keen interest in the optimal management of neurological conditions in resource-limited settings. Based in southern Africa for half of the year, she has first-hand experience in the management of such conditions and, in particular, of epilepsy, which is one of her main areas of interest (Figure 1).

Gretchen is also one of the guest editors for the *BMC Medicine *article collection, *Medicine for Global Health*. Here we talk to Gretchen about her personal views on the challenges of epilepsy management from a global perspective.

## 1) How is epilepsy currently managed in resource-limited settings, and what are the difficulties with its current management in these settings?

There isn't really one answer to that as care in different resource-limited settings is very dependent upon care-seeking behaviors, the expertise of existing healthcare personnel (especially at the primary healthcare level) and drug availability. Where there are active community-based efforts to bring epilepsy and people with epilepsy "out of the shadows", care-seeking will be primarily limited by patient resources and transport infrastructure. But if people with epilepsy and their families misunderstand the nature of seizures, epilepsy may never be brought to the formal healthcare sector for assessment; people will see traditional healers. Primary healthcare workers need to have adequate training in the basics of diagnosis and treatment with recourse to referral for higher level evaluation in complicated cases. Without trained primary healthcare staff, most patients will not get treated at all. The development of training programs appropriate for a specific resource-limited setting really needs to be guided by physician-level experts who know the healthcare environment, and they also need to know the local culture and the local epidemiology of disease. And even if all of these are in place, if antiepileptic drug supply lines are inefficient or inconsistent, adequate management isn't possible. It is a chain of "necessary but not sufficient" factors and each of these likely vary from country-to-country and possibly even within country in resource-limited settings.

## 2) What are the main differences with epilepsy management in resource-limited settings as compared to high income countries?

Many of the things people with epilepsy struggle with are universal. Women with epilepsy worry about the impact of their condition and their medications on their offspring. Children with epilepsy struggle to be accepted and succeed in school. Adolescents with epilepsy fear rejection from their peers. These are pretty common issues. Medication side effects are problematic whether you have two drugs to choose from or ten. Comorbid and often untreated depression and anxiety degrade quality of life for people with epilepsy wherever you are. Access to medications is more problematic in resource-limited settings - but if you've worked with the employed but uninsured in the US, you know this is NOT a problem unique to "resource-limited settings".

What is different are the consequences of the medical, social and economic burden of epilepsy in settings where people are already on the cusp of survival. If you cook over an open flame, have poor seizure control because medication access is intermittent and live where contagion fears are common, a generalized seizure can mean extensive burns and death or a lifetime physical disability. For women with epilepsy, spousal or familial abandonment due to misconceptions regarding epilepsy and in the absence of a social safety net results in vulnerability to sexual predators and marginal survival at best. If educational opportunities are lost due to epilepsy, those with childhood onset face lifetime limitations in their personal capacity even if their epilepsy remits by adulthood.

## 3) Have there been any implications for neurology in resource-limited settings in terms of results from the Global Burden of Disease (GBD) study?

The most impressive outcome for the epilepsy field in the Global Burden 2010 work was the value assigned to having epilepsy in the Disability-Adjusted Life Year (DALY). Previous DALY assignments were made largely by expert groups and their opinions. In the GBD2010 survey of almost 14,000 people from a range of global settings (the US, Bangladesh, Indonesia, Peru and Tanzania), severe epilepsy was rated as having a DALY of 0.657. This was almost the highest rated disability assessed - even untreated AIDS without antiretroviral medications had a less burdensome DALY at 0.547, remembering that a DALY of 1 essentially means 'dead'.

## 4) Where do you think work should be done in this area to improve clinical outcome of those affected?

Geographically "where"? There is doubtless room for improvement everywhere. Even in economically developed settings there are substantial healthcare disparities being discovered among people with epilepsy.

In resource-limited settings, I think we need to have a dual research agenda - one aimed at improving the lives of people with epilepsy through improved healthcare services and community-oriented efforts to diminish the social and economic impact of the condition. The second area for work should be on the prevention of epilepsy. There are ample data pointing at some of the more common causes of epilepsy in these environments and many of the causes are potentially modifiable, for example, better management of acute cerebral malaria, and better management of antenatal risk factors for perinatal brain injury.

## 5) Where can I find more information?

Ding D, Wang W, Wu J, Yang H, Li S, Dai X, Yang B, Wang T, Yuan C, Ma G, Bell GS, Kwan P, de Boer HM, Hong Z, Sander JW: **Premature mortality risk in people with convulsive epilepsy: long follow-up of a cohort in rural China**. *Epilepsia *2012. [Epub ahead of print].

Birbeck G, Chomba E, Atadzhanov M, Mbewe E, Haworth A: **The social and economic impact of epilepsy in Zambia: a cross-sectional study**. *Lancet Neurol *2007, **6**:39-44.

Salomon JA, Vos T, Murray CJ: **Disability weights for vision disorders in Global Burden of Disease study - Authors' reply**. *Lancet *2013, **381**:23-24.

Kroner BL, Fahimi M, Kenyon A, Thurman DJ, Gaillard WD: **Racial and socioeconomic disparities in epilepsy in the District of Columbia**. *Epilepsy Res *2013, **103**:279-287.

Bautista RE, Jain D: **Detecting health disparities among Caucasians and African-Americans with epilepsy**. *Epilepsy Behav *2011, **20**:52-56.

Birbeck GL, Molyneux ME, Kaplan PW, Seydel KB, Chimalizeni YF, Kawaza K, Taylor TE: **Blantyre Malaria Project Epilepsy Study (BMPES) of neurological outcomes in retinopathy-positive paediatric cerebral malaria survivors: a prospective cohort study**. *Lancet Neurol *2010, **9**:1173-1181.

Ngugi AK, Bottomley C, Kleinschmidt I, Wagner RG, Kakooza-Mwesige A, Ae-Ngibise K, Owusu-Agyei S, Masanja H, Kamuyu G, Odhiambo R, Chengo E, Sander JW, Newton CR; SEEDS group: **Prevalence of active convulsive epilepsy in sub-Saharan Africa and associated risk factors: cross-sectional and case-control studies**. *Lancet Neurol *2013, **12**:253-263.

## Pre-publication history

The pre-publication history for this paper can be accessed here:

http://www.biomedcentral.com/1741-7015/11/70/prepub

